# Measuring sexual violence stigma in humanitarian contexts: assessment of scale psychometric properties and validity with female sexual violence survivors from Somalia and Syria

**DOI:** 10.1186/s13031-021-00431-z

**Published:** 2021-12-24

**Authors:** Sarah M. Murray, Molly E. Lasater, Marie-France Guimond, Ohemaa Poku, Rashelle Musci, Manal Al-Fataftah, Lilian Kasina, Mercy Lwambi, Asma Salaimeh, Kathryn Falb

**Affiliations:** 1 Department of Mental Health, Johns Hopkins Bloomberg School of Public Health, 624 N Broadway Street, Baltimore, MD 21205 USA; 2 Airbel Impact Lab, International Rescue Committee, New York, NY USA; 3 Women Protection and Empowerment Program, International Rescue Committee, Amman, Jordan; 4 Design Monitoring and Evaluation Unit, International Rescue Committee, Nairobi, Kenya; 5 Women’s Protection and Empowerment Program, International Rescue Committee, Nairobi, Kenya; 6 Monitoring, Evaluation, Accountability and Learning Unit, International Rescue Committee, Amman, Jordan; 7 Airbel Impact Lab, International Rescue Committee, Washington, DC USA

**Keywords:** Stigma, Sexual violence, Measurement, Psychometrics, Item response theory, Syria, Somalia

## Abstract

**Background:**

Valid measures of sexual violence stigma that can be readily incorporated into program monitoring and evaluation systems are needed to strengthen gender-based violence (GBV) services in humanitarian emergencies. This study sought to assess the psychometric properties, construct validity, and measurement invariance of sexual violence stigma scales among female Somali GBV survivors in Kenya and Syrian GBV survivors in Jordan to identify an abbreviated scale that could be used across humanitarian contexts.

**Methods:**

We administered measures of sexual violence stigma to 209 female survivors of sexual violence aged 15 and older in Kenya and Jordan. Exploratory factor analysis was used to assess the underlying latent structure, and Item Response Theory was used to estimate item difficulty and discrimination parameters to guide efforts to shorten the scales. Differential item functioning (DIF) by site was assessed using Multiple Indicators, Multiple Causes models. Construct validity of the sexual violence stigma scales was assessed by estimating correlations with functional impairment, depression, and disability.

**Results:**

The sexual violence stigma measure exhibited distinct factor structures among Somali and Syrian GBV survivors. Among Somali survivors, a two-factor model with separate felt (10 items) and enacted (4 items) stigma constructs was identified, with scales for both domains exhibiting good internal consistency (Cronbach’s alpha = 0.93 and 0.88, respectively). In Jordan, a single factor solution was uncovered for a 15-item stigma scale with good internal consistency (alpha = 0.86). The shortened core sexual stigma scale consisting of the 4 items that did not exhibit DIF had a Cronbach’s alpha of 0.82 in Kenya and 0.81 in Jordan. The felt stigma scale in Kenya, the full stigma scale in Jordan, and abbreviated core stigma scales in both countries were meaningfully correlated with depression, while correlations with functional impairment were weaker and inconsistent across scales.

**Conclusions:**

An abbreviated core set of invariant perceived and internalized sexual violence stigma items demonstrated evidence of construct validity in two diverse settings. The ability of this measure to be efficiently administered as a part of routine program monitoring and evaluation activities, with the potential addition of items from a measurement bank to improve contextual relevance, can facilitate improvements in the delivery and quality of gender-based violence programs in humanitarian emergencies.

**Supplementary Information:**

The online version contains supplementary material available at 10.1186/s13031-021-00431-z.

## Background

Gender-based violence (GBV), broadly defined to include physical, sexual, economic, or psychological violent acts, threats or coercion that are rooted in gender norms or inequality, is widely experienced by women living in diverse settings around the globe [1–4]. The past year prevalence of physical or sexual intimate partner violence among ever-partnered women aged 15–49 has been found to range from 5 to 40% across low- and middle-income counties (LMIC), with a greater prevalence observed among lower-income women [1]. In addition, a meta-regression of data from more than 50 countries identified the global prevalence of lifetime non-partner sexual violence, another form of GBV, as 7.2% [5].

In humanitarian emergencies, as many as one in five women report an experience of sexual violence by a partner or non-partner, possibly due to both the use of sexual violence as a tool of war and increases in perpetration due to conflict-related damage to protective family, social, and institutional structures (e.g., law enforcement) [6]. A recent study from conflict-affected Southern Sudan found that women reported a prevalence of lifetime experiences of non-partner sexual violence that was four times the global mean [7]. Women exposed to conflict are also more likely to report physical and/or sexual intimate partner violence (IPV) [8], with qualitative research from multiple refugee camp settings suggesting the importance of disruption in gender roles, changes to family structure, and men’s substance use in affecting refugee women’s risk [9]. Conflict has also been found to be associated with both men’s and women’s acceptance of physical intimate partner violence at the country level [10]. Therefore, responses to gender-based violence in humanitarian contexts must be robust to addressing both violence committed by partners and non-partners, and violence that may be sexual and/or physical.

Serious health consequences of GBV can be physical, including injury, fistulae, and sexually transmitted infections [11–13], as well as psychological, including post-traumatic stress disorder (PTSD), depression, and suicidality [12, 14–18]. Sexual violence in conflict-affected settings has been found to negatively affect survivors’ relationships with their community, family, and partners due to stigma surrounding the experience and its consequences [14, 19–23]. Stigma experiences can include the perception of maltreatment by others (perceived stigma) and/or having been subjected to harmful behaviors or acts of discrimination (enacted stigma) on account of being a survivor of sexual violence [24]. Survivors may also internalize stigma associated with experiences of violence, developing feelings of shame and withdrawing from others [14, 19, 21, 25]. Further, experiences of stigma may mediate the impact of sexual violence on women’s wellbeing [23]. One possible pathway by which stigma may impact overall health is by discouraging survivors from seeking out formal and informal support. For instance, in northern Uganda, 87% of survivors of sexual violence reported perceiving stigma and discrimination as a barrier to seeking mental health services [26]. As children born from sexual violence are often rejected or experience other forms of enacted stigma [22], sexual violence stigma may have intergenerational or compounding effects for women. For instance, in northern Uganda, women who had a child as a result of sexual violence experienced greater odds of perceiving and experiencing stigma or discrimination, of having a poor relationship with their community, and of reporting poorer mental health and general functioning than those with no children [27].

Several humanitarian organizations have compiled guidelines outlining how to provide quality, comprehensive care to survivors of GBV, including women who have experienced sexual violence. To facilitate more effective GBV programming in humanitarian emergencies, the United Nations Population Fund (UNFPA) put forth a set of minimum standards in 2015 for how humanitarian actors should design, coordinate, and implement GBV intervention activities related to mitigation, prevention and response [28]. Interventions integrated into the minimum standards for GBV programming included rape-related clinical care offered within health services, mental health and psychosocial support, legal assistance, socio-economic empowerment services, and coordinated referral systems for other needs. Furthermore, the UNFPA guidelines recommend that these services be implemented in the context of broader measures that ensure the safety and security of survivors throughout the course of humanitarian response in an integrated fashion [28]. Establishment of safe spaces, distribution of dignity kits (i.e., packs of hygiene materials that may include soap, underwear, menstrual pads, etc.) and/or cash transfers, support for livelihoods, and broader efforts at changing gender norms and acceptability of violence are also recommended as part of comprehensive programming within the Interagency Minimum Standards [29]. Each of these strategies has the potential to affect and be affected by sexual violence stigma, which is recognized as a barrier to accessing care and as a factor to consider in the provision of care within these guidelines.

UNFPA also includes the collection and use of data to monitor and evaluate programs that address GBV in emergencies as an operational standard, echoing others’ assertion that there is an acute need for rigorous monitoring and evaluation (M&E) of humanitarian response programs [28, 30, 31]. M&E facilitates programmatic learning, helps ensure that services delivered are of high quality, and keeps programs accountable to stakeholders such as program participants and funder [30]. Measurement of psychosocial wellbeing as a part of M&E activities during emergencies is of particular importance to ensure the reasonable and effective use of scarce resources and prevent doing harm to vulnerable populations [32]. Tracking psychosocial responses to GBV programs not only allows organizations to determine if they are meaningfully impacting women’s well-being, but to identify survivors who are not responding to more general promotive and preventative psychosocial services and may require additional intensive mental health care services. Given how commonly and meaningfully stigma is described as a problem among sexual violence survivors, its links to health outcomes, and the potential for programs to either improve or exacerbate stigma unintentionally, it is an essential psychosocial outcome to monitor in GBV programming. However, given the need to track multiple health and social outcomes in programs while minimizing burden for women seeking services, having measures that are not only acceptable and valid, but brief, is of particular importance.

Assessing the impact of GBV programs on a survivors’ perception of stigma requires comprehensive, valid, and reliable measures. The topic of stigma may arise in response to open-ended questions and discussions that take place as a part of case management, but quantitative measures allow service providers to track stigma among survivors in a standardized way. While an increasing number of measures have been validated to assess symptomatology of mental health disorders in diverse humanitarian emergency contexts [33], the psychometrics and validity of measures of social wellbeing in general, and stigma in particular, are less commonly evaluated. Further, while some measures have been developed or adapted to assess stigma related to sexual violence or intimate partner violence, the utility, performance, and validity of these measures across cultures and contexts for widespread use in program monitoring and research have not been explored [14, 34, 35].

Previously, two measures of sexual violence-related stigma were developed for use with women in the Democratic Republic of Congo (DRC): felt stigma, which included a combination of items related to women’s perceptions of different treatment by others and internalization of stigma; and enacted stigma, which assessed experiences of discrimination or mistreatment. Both stigma scales demonstrated evidence of construct validity and were found to have adequate internal consistency [21]. The felt stigma scale was then successfully used to assess the impact of group Cognitive Processing Therapy (CPT) and a Village Savings and Loans (VSLA) program for survivors of sexual violence within the same setting and population in two randomized controlled trials [36, 37]. In the current study, we aimed to assess whether these scales could be adapted to measure the impact of GBV response programming in other humanitarian emergency contexts as part of an effort to expand M&E of gender-based violence programming to include broader social and functional-related outcomes. Specifically, in the following, we present findings from piloting these measures of sexual violence stigma in Jordan and Kenya as a part of routine M&E services to assess their psychometric properties and construct validity across these two diverse settings. We also explored if these measures could be abbreviated to enhance their potential for implementation within programming and developed recommendations for their use within gender-based violence services being delivered in diverse conflict-affected settings going forward.

## Methods

Data was collected by the International Rescue Committee (IRC) between March and May 2018 from adult women and older adolescent girls accessing GBV case management services in two locations: Jordan, specifically through Women and Girls’ Safe Spaces housed in Mafraq, Irbid, east Amman, Zarqa, and Ramtha, as well as mobile delivered support services; and the Dadaab refugee complex in Kenya, where IRC was running Women’s Protection and Empowerment one-stop centers. In 2020, IRC provided case management services to more than 1100 survivors of GBV in Jordan and nearly 850 in Kenya. More than a million Syrians are estimated as being displaced in Jordan on account of the war that began in 2011, with the vast majority of the more than 600,000 refugees registered with UNHCR living outside of camp settings [38]. A recent systematic review found that while the exact prevalence of sexual violence among displaced Syrian women is unclear, they are at risk for multiple forms of violence including early and/or forced marriage, and approximately one third of Syrian refugees surveyed in Lebanon reported exposure to conflict-related violence [39, 40]. Since the early 1990s when the Dadaab camp was established, Somali refugees have fled to Kenya on account of civil war, drought, and famine [41]. Conflict has occurred within the camp and with the host community as the camp is overpopulated and resources are scarce [42]. A survey of Somali women accessing GBV services in Dadaab found that nearly half reported past year intimate partner violence, and more than a third reported past year non-partner violence, 16% of which was sexual [41].

After receiving between three to five sessions of GBV survivor case management services, potential participants were asked if they would like to participate in a survey. If so, a separate GBV case manager conducted oral informed consent procedures with the woman if eligible and administered the survey to the woman in a private setting, with interviewers trained to re-assess privacy at multiple points in the interview and pause or discontinue if privacy was not ensured. Interviewers were also trained to remind women that they could skip questions and to pause or stop the interview if a participant became distressed. Interviewers were equipped to refer women to local psychosocial support if appropriate, in addition to continuing their case management services. To be eligible, women and older adolescent girls had to be 15–65 years of age with no observable cognitive difficulties. IRC did not specifically assess if women had been displaced given sensitivities around documentation, though the location and targeting of these services was designed to cater specifically to refugees. All data were collected on tablets via Kobo and uploaded on a weekly basis to a secure server.

All study participants provided informed consent. Parental consent was waived for older adolescent girls (ages 15–17) who were treated as adults within the study. This request was made to the ethical review boards in order to prioritize confidentiality and reduce risk of furthering stigma in the event that an adolescent's caregiver was unaware of her accessing services for violence, as treatment for girls 15 and older in GBV case management services is self-determined. Ethical review for the original research data collection was provided by the ethical review board of the International Rescue Committee, the Kenya Medical Research Institute (KEMRI), and a community advisory board in Jordan. The Johns Hopkins Bloomberg School of Public Health Institutional Review Board reviewed the secondary analysis of deidentified data collected by IRC and exempted the analysis as non-human subjects research.

### Measures

The measures administered by IRC and included in the final study dataset are detailed below.

#### Socio-demographics

Women reported their age, marital status, whether they were currently living with their partner, years of education completed, number of people living in their home, the number of children for whom they are responsible, and the number of years they had been living in the current area. In addition, women also indicated if they were currently pregnant or suffering from a disability.

#### Functioning

A measure of daily functional impairment developed originally among survivors of sexual violence in the Eastern DRC was adapted to the two local study contexts. In addition to changing the exact wording of items, 7 items were added to the scale that represented common tasks of living from these two new locations not already reflected in the existing scale based on the results of six focus groups (three in each country) with IRC women’s protection and empowerment social workers, refugee outreach workers, and response officers, as well as individuals from community-based partner organizations in each country. These focus groups also included the selection and adaption of a pictural aid for the rating scale. One item specific to the school context was excluded due to the number of individuals who indicated this was not applicable given the age of the population. For a total of 26 items, respondents indicated how much difficulty they had completing a task in the past 4 weeks on a scale from 0 (not difficult at all) to 4 (so difficult that you often cannot do it) accompanied by a pictorial aid. Participant item responses were averaged to create a mean score. In the case that a respondent indicated the item was not applicable to them, simple mean imputation was used. Cronbach’s alpha for the scale indicated acceptable internal consistency (α = 0.88).

#### Depression

The 9-item Patient Health Questionnaire (PHQ-9) was selected to assess depression due to its prior use and demonstration of acceptable internal consistency with both populations [43–45]. Respondents indicated the number of days over the past 4 weeks they had experienced each depression-related symptom on a 4-point Likert scale from 0 (not at all) to 3 (nearly every day). Cronbach’s alpha for the PHQ-9 in this sample was 0.87. In addition, a 10th item on this scale asks participants to indicate how difficult it has been completing work, taking care of things at home, or getting along with others due to these problems: not difficult at all, somewhat, very, or extremely difficult. The score for all items was averaged to create a scale score that could range from 0 to 3.

#### Sexual violence stigma

A total of 17 sexual violence stigma items were included on the study survey. An 8-item scale for assessing a combination of perceived and internalized stigma among survivors of sexual violence in the DRC was adapted for use in the current study [21]. In addition, four new items were added (“feeling like your family gazes at you like they are blaming you,” “feeling like community members gaze at you like they are blaming you,” “feeling like friends and classmates at school gaze at you like they are blaming you,” and “wanting to change the way you dress”) and one item that was dropped during the original scale development process (“blaming self for things”) was changed to “blaming self for past events” and included. Accompanied by a pictorial aid, women indicated on a Likert scale of 0–3 how often they had the thought or feeling in the past 4 weeks: not at all, a little bit, a moderate amount, or a lot. In addition, four enacted stigma items were included in the study measure based on the prior stigma scale development in DRC [21]: having been abandoned or thrown out of one’s home, been rejected by their family, rejected by their intimate partner, or being forced to live away from your husband due to the violence or trauma a woman experienced. These items loaded onto their own distinct factor termed enacted stigma in the prior study. For each of these items, women indicated whether they had ever had this experience, yes or no.

#### Disability

The 12-item WHO Disability Assessment Schedule (WHODAS 2.0) was included to measure disability. For each item, individuals were asked to think about the activity and indicate how difficult it was to carry out in the last 4 weeks on a Likert-type scale that ranged from 0 “Not difficult at all” to 4 “So difficult that you often cannot do it.” Average scores were calculated for each participant. Cronbach’s alpha for the WHO-DAS 2.0 was 0.83, indicating adequate internal consistency in these samples.

### Analysis

Exploratory data analysis included assessment of missingness, variable distributions, and measures of central tendency and dispersion for all measures and sociodemographic variables overall and by country. Stigma items with a high degree of missingness or limited response distributions were considered for removal from the scales.

Exploratory factor analysis (EFA) was then used to assess the underlying factor structure of the stigma items. EFAs for these items were conducted separately for data from women in Jordan and Kenya. A polychoric correlation matrix was used to account for the ordinal nature of the indicators. To select the number of factors for inclusion in the EFA, a Principal Components Analysis (PCA) was first conducted. The number of eigenvalues over 1, percentage of variance explained, and examination of a scree plot were considered in selecting the number of factors to include. In addition, the results of a parallel analysis run using an underlying Pearson correlation matrix were also examined. An EFA was then run, again using a polychoric correlation matrix and a weighted least squares estimator. Promax rotation was implemented to aid in interpretation of multifactorial models. Cronbach’s alpha was also calculated to assess internal consistency of each scale as determined from the EFAs. While there is no one rule for considering a strong factor loading [46], we considered items to meaningfully load on a factor if the loading was ≥ 0.4. However, revisions to scales were guided by item loadings in addition to item-rest correlations, content validity, and improvements to Cronbach’s alpha ≥ 0.001.

An item response theory (IRT) analysis was then used to shorten the stigma scales (refined based on the EFA results) to improve their utility in service settings in humanitarian emergencies. Specifically, the discrimination and location parameters for each item were estimated in a graded response model [47, 48]. In a graded response model, item location parameters are estimated (one less parameter than the number of response categories) as the point on the latent trait (i.e., stigma in this analysis) at which there is a 50% probability of selecting a given ordinal response option or higher [49, 50]. A location parameter is therefore synonymous with item difficulty, and it represents the point on the latent trait at which discrimination is the highest for a given response on a given item. Discrimination is calculated as the slope on the item characteristic curve at the location parameter. We prioritized the selection of stigma items with high discrimination > 1.35 and varying levels of difficulty for inclusion in the abbreviated scale [49].

In addition, we explored differential item functioning (DIF) of these shortened, refined scales across the two countries by estimating Multiple Indicators, Multiple Causes Models (MIMIC). Differential item functioning across sub-groups (in this case, country of national origin or location) occurs when individuals with the same value of the underlying trait (i.e., stigma) respond differently to an item due to the uneven distribution of another characteristic across these subgroups [51]. To select items that potentially exhibited DIF, we first estimated the stigma measurement model with country as a predictor of the latent stigma construct, and one by one allowed each retained stigma item to also be directly predicted by the country variable outside of the relationship with the latent construct [52]. We examined the Bonferroni adjusted *p* values (the *p* value multiplied by the number of tests performed) of the country to stigma item paths and considered any statistically significant item (*p* < 0.05) to have DIF potential. We then tested a series of constrained models versus a full model where all items identified as potentially exhibiting DIF were regressed on the country variable. The constrained models were generated by removing the path from country to one potential DIF item at a time, i.e., treating that item as invariant. A likelihood ratio test for nested models was then performed comparing each constrained model to the full model. For any test where the constrained model produced a significant *p* value (< 0.05), indicating significantly worse fit, that item was determined to exhibit DIF (i.e., the model where country was allowed to directly predict the item response fit significantly better than when it was not) [52]. Again, *p* values were Bonferroni adjusted (multiplied by the number of comparisons between full and constrained models performed).

For the resulting stigma scales, scores were calculated as an average for each participant, with simple mean imputation used for item-level missingness (all items < 5% missing, except “rejected by your intimate partner” which had missingness = 8%) and no average score given for any individual missing 40% or more of the items on a given scale. Construct validity was assessed by estimating Pearson correlations between the stigma scale scores and several measures (described above) of constructs from the nomological network of sexual violence stigma: functional impairment, depression, and disability. We hypothesized that all forms of sexual violence stigma would be positively correlated with symptoms of depression and functional impairment, given observed associations between these domains and different manifestations of stigma related to a variety of attributes, including sexual violence, in the literature from low and middle-income countries [14, 23, 53]. We had the same hypothesis for associations between stigma and disability on account of several factors: the WHO-DAS’ emphasis on daily functioning; employment of an intersectional perspective that might suggest women with a disability are more vulnerable to experiencing stigma or may be more stigma aware; and, literature showing greater violence experiences and poorer mental health among Somali refugee women who reported a disability compared to those who did not [54]. Analyses were conducted using Stata, version 15 [55], except for the EFAs, IRT and MIMIC models, which were implemented using Mplus, version 8 [56].

## Results

### Sample characteristics

A total of 217 women were approached, of whom 8 individuals declined participation and 209 (96.3%) were consented into the study. One consented woman did not complete the study questionnaire; thus, 208 (99.5%) women contributed data to these analyses. The sample included 100 women from Kenya and 108 from Jordan. Sociodemographic characteristics of the sample are presented in Table 1. While women’s ages ranged from 15 to 65 years old, only 6 participants (2.9%) were under age 18. Approximately half of the women (49.0%) were married and 4 out of 5 (78.8%) married women were living with their partner. In comparing women between the two sites, women in Jordan were statistically significantly older (34.6 vs. 31.4 years old) and more likely to be married (62% vs. 35%) than women in Kenya. Women in Jordan also reported significantly more education on average than women in Kenya (8.0 vs. 2.2 years), while women in Kenya were living in homes with a greater number of people (7.3 vs. 5.5), were caring for more children (4.4 vs. 3.0) and were more likely to be pregnant at the time of survey (22.0% vs. 8.4%) than women in Jordan. Women in Kenya were also significantly more likely to report having a disability (19.0%) than women in Jordan (5.6%). Women in Jordan had been living in their current area on average for a shorter period than women in Kenya (5.5 vs. 9.5 years).

**Table 1:** Sociodemographic characteristics of study participants

	Jordan (n = 108)	Kenya (n = 100)
Age, mean (sd)*	34.6 (8.6)	31.4 (9.1)
Married, n (%)*	67 (62.0)	35 (35.0)
Lives with partner (if married), n (%)	52 (80.0)	26 (76.5)
Years of education, mean (sd)*	8.0 (4.0)	2.2 (4.0)
Number of people living in home, mean (sd)*	5.5 (2.7)	7.3 (3.8)
Number of children responsible for, mean (sd)*	3.0 (1.9)	4.4 (3.2)
Pregnant, n (%)	9 (8.4)	22 (22.0)
Has a current disability, n (%)*	6 (5.6)	19 (19.0)
Years lived in current location, mean (sd)*	5.5 (5.3)	9.5 (6.5)
PHQ-9 score for depression, mean (sd)*	1.2 (0.6)	0.9 (0.8)
WHO-DAS score for disability, mean (sd)*	1.2 (0.6)	0.7 (0.6)
Functional impairment, mean (sd)*	1.2 (0.6)	0.6 (0.5)

Missingness was less than 5% on all variables. Education and number of children responsible for was missing for 8 women (3.8%); functional impairment for six women (2.9%); number of people living in the home and current pregnancy for 2 women (1.0%), current disability status for one woman (0.5%); average depression score for one woman (0.5%); Years lived in current place was missing or reported as “don’t know” for 33 women (15.9%). Living with partner was missing for 3 (2.9%) women who were married (n = 102)

*p value < 0.05 for *t* test or rank sum test of difference in means by country or chi-squared test in frequencies by country

The average depression score at baseline was 1.05 in the total sample, and relatively higher in Jordan (1.2) versus Kenya (0.9). Disability scores were also substantially higher in Jordan (mean = 1.2) than Kenya (mean = 0.7), as were functional impairment scores (mean = 1.2 in Jordan and mean = 0.6 in Kenya).

### Stigma

**Fig. 1:**
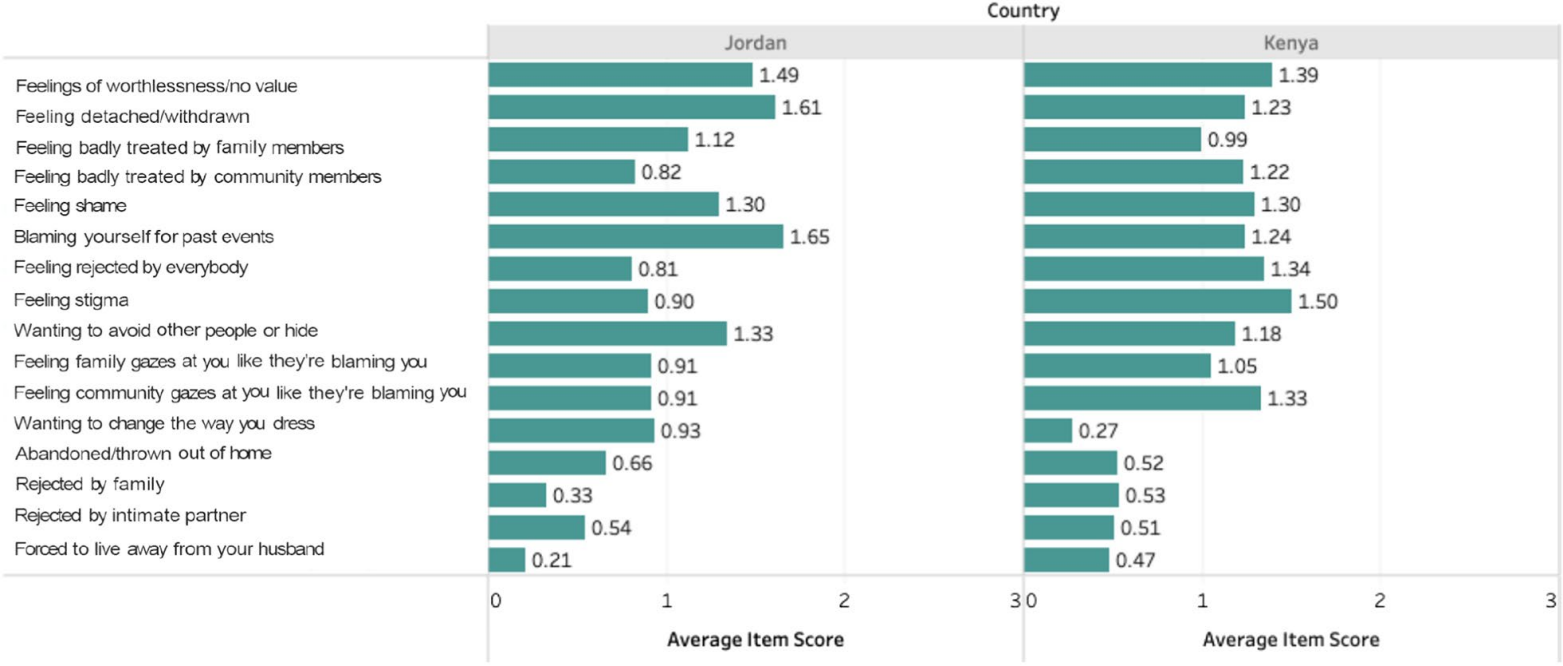
Average stigma item score by country

One stigma item, “feeling like friends and classmates at school gaze at you like they are blaming you,” was dropped prior to analysis given the low number of girls under 18 in our sample who were enrolled in school and could respond. Figure 1 displays the average item score for each stigma item by country and demonstrates differences in the most common types of stigma experiences across the two settings. The most frequent stigma experiences in Jordan included “feeling detached or withdrawn from others,” “blaming yourself for past events,” and having “feelings of worthlessness or no value,” while the least commonly endorsed experiences were feeling badly treated by family and feeling rejected by everybody. In Kenya, the most frequently endorsed stigma experiences were feeling stigma, feelings of worthlessness or having no value, feeling rejected by everybody, and feeling like the community gazes at you like they are blaming you. The item “wanting to change the way you dress” was rarely endorsed in Kenya.

Among women in Kenya, the PCA produced 4 eigenvalues over 1 (8.65, 3.05, 1.36, 1.11) that collectively explained 88.9% of the variance. The first eigenvalue alone explained 54.1% of the variance. The parallel analysis suggested a 2-factor solution to be optimal, the results of which are presented in Table 2. Factor 1 was termed “Felt Stigma” with all perceived and internalized stigma items loading together on this factor. Factor 2 was termed “Enacted stigma” as all the enacted stigma items loading together on this second factor. Only the item “wanting to change the way you dress” failed to load above 0.4 on either factor, and no item loaded at or above 0.4 on more than one factor. The resulting 11-item Felt Stigma scale and 4-item Enacted Stigma scale had a Cronbach’s alpha of 0.92 and 0.88, respectively. Removing the item “feeling badly treated by family members” from the Felt Stigma scale resulted in an improvement of the alpha of 0.005, and this item was therefore dropped to create a 10-item scale going forward.

**Table 2:** Two factor EFA results for the sample of women residing in Kenya (n = 100)

Stigma items	Felt stigma loading	Enacted stigma loading	Residual variance
Feelings of worthlessness, of having no value *(i)*	0.95*	− 0.38*	0.21
Feeling detached or withdrawn from others *(i)*	0.96*	− 0.31*	0.19
Feeling badly treated by family members *(p)*^a^	0.53*	0.23	0.58
Feeling badly treated by community members *(p)*	0.99*	− 0.25*	0.14
Feeling shame *(i)*	0.91*	0.01	0.16
Blaming yourself for past events *(i)*	0.77*	0.14	0.31
Feeling rejected by everybody *(i)*	0.94*	− 0.07	0.16
Feeling stigma *(p)*	0.91*	0.04	0.15
Wanting to avoid others or hide *(i)*	0.82*	0.09	0.29
Feeling like your family gazes at you like they are blaming you *(p)*	0.63*	0.25	0.43
Feeling like community members gaze at you like they are blaming you *(p)*	0.94*	0.03	0.10
Wanting to change the way you dress *(i)*	0.29	0.01	0.91
Been abandoned/thrown out of your home *(e)*	0.05	0.90*	0.16
Rejected by your family because of trauma you experienced *(e)*	0.19	0.84*	0.15
Rejected by your intimate partner because of trauma you experienced *(e)*	0.002	0.96*	0.08
Forced to live away from husband because of violence you experienced *(e)*	− 0.03	0.95*	0.13

^a^Though this item loaded above 0.4, dropping it from the scale improved the alpha by ≥ 0.001, and thus it was not retained

*Loading significant at *p* < 0.05; e = enacted stigma item; i = internalized stigma item; p = perceived stigma item; Bold indicates the item was retained for future analyses

In Jordan, the PCA also produced 4 eigenvalues over 1 (6.90, 1.96, 1.36, 1.22) that collectively explained 71.5% of the variance. The first eigenvalue alone explained 43.1% of the variance. The parallel analysis indicated a one factor solution to be optimal. Results of a 1- and 2-factor EFA (for comparability with the Kenya results) of data from the woman in Jordan is presented in Table 3.

**Table 3:** One and two factor EFA results for the sample of women residing in Jordan (n = 108)

Stigma items	1 factor solution		2 factor solution		
	All stigma loading	Residual variance	Internalized stigma loading	Community or family stigma loading	Residual variance
Feelings of worthlessness, of having no value *(i)*	0.64*	0.60	0.78*	− 0.07	0.44
Feeling detached or withdrawn from others *(i)*	0.68*	0.54	0.77*	0.00	0.41
Feeling badly treated by family members *(p)*	0.50*	0.75	0.25	0.33*	0.74
Feeling badly treated by community members *(p)*	0.55*	0.70	− 0.10	0.72*	0.55
Feeling shame *(i)*	0.51*	0.75	0.61*	− 0.04	0.65
Blaming yourself for past events *(i)*	0.64*	0.59	0.68*	0.05	0.51
Feeling rejected by everybody *(i)*	0.79*	0.38	0.32*	0.58*	0.37
Feeling stigma *(p)*	0.81*	0.34	0.54*	0.40*	0.32
Wanting to avoid others or hide *(i)*	0.76*	0.43	0.73*	0.13	0.35
Feeling like your family gazes at you like they are blaming you *(p)*	0.79*	0.37	0.27*	0.62*	0.35
Feeling like community members gaze at you like they are blaming you *(p)*	0.76*	0.43	− 0.01	0.88*	0.23
Wanting to change the way you dress *(i)*	0.20*	0.96	0.02	0.22	0.95
Been abandoned/thrown out of your home *(e)*	0.54*	0.70	0.08	0.56*	0.64
Rejected by your family because of trauma you experienced *(e)*	0.63*	0.60	0.06	0.66*	0.52
Rejected by your intimate partner because of trauma you experienced *(e)*	0.58*	0.67	− 0.06	0.73*	0.52
Forced to live away from husband because of violence you experienced *(e)*	0.45*	0.80	0.18	0.34*	0.79

*Loading significant at p < 0.05; e = enacted stigma item; i = internalized stigma item; p = perceived stigma item; Bold indicates the item was retained for future analyses

In the two-factor solution from Jordan, items on worthlessness, detachment and isolation, shame, and blame loaded together. We termed this factor “Internalized Stigma.” The perceived stigma items “feeling badly treated by community members,” “feeling rejected by everybody,” “feeling like family and community blame you,” and three of four of the enacted stigma items all loaded together on a second factor which we termed “Community and Family Stigma.” “Feeling badly treated by family members,” “wanting to change the way you dress,” and “being forced to live away from one’s husband because of the violence experienced” did not load above 0.4 on either factor, while “feeling stigma” cross-loaded at or above 0.4 on both factors. After dropping items that did not load above 0.4 on either factor or cross-loaded at or above 0.4 on both factors, Cronbach’s alpha for the 5-item Internalized Stigma scale was 0.78 and was 0.80 for the 7-item Community and Family stigma scale. In the one-factor EFA, all items loaded above 0.4 except “wanting to change the way you dress.” Cronbach’s alpha for the total combined 15-item scale (dropping only “wanting to change the way you dress”) was 0.86.

On account of the different factor structures identified in the two countries, we performed the IRT analysis to shorten the stigma measure on the longer of the two scales developed in the sample of women from Kenya (the 10-item Felt Stigma scale; Table 5) and the full All Stigma 15-item scale in Jordan (Table 6). In Kenya, the IRT estimated discrimination parameter for all Felt Stigma items was greater than 1.35 (Table 4). In Jordan, all enacted stigma items, two perceived stigma items (“feeling badly treated by family members” and “feeling badly treated by community members”), and two internalized stigma items (“feelings of worthlessness, of having no value” and “feeling shame”) had a discrimination that was less than 1.35 (Table 5).

**Table 4:** Item response analysis of retained felt stigma items in the sample from Kenya (n = 100)

Felt Stigma items	Discrimination	Difficulty		
		≥ 1	≥ 2	= 3
Feelings of worthlessness, of having no value *(i)*	2.17	− 0.30	0.24	0.48
**Feeling detached or withdrawn from others *(i)***	2.38	− 0.23	0.49	0.63
Feeling badly treated by community members *(p)*	4.59	0.03	0.37	0.50
Feeling shame *(i)*	4.06	0.09	0.27	0.43
**Blaming yourself for past events *(i)***	2.53	0.02	0.41	0.53
**Feeling rejected by everybody *(p)***	4.47	− 0.003	0.26	0.40
**Feeling stigma *(p)***	4.37	− 0.13	0.13	0.30
**Wanting to avoid others or hide *(i)***	2.72	0.04	0.46	0.61
**Feeling like your family gazes at you like they are blaming you *(p)***	1.60	0.27	0.60	0.88
**Feeling like community members gaze at you like they are blaming you *(p)***	4.97	− 0.002	0.25	0.47

Suggested items for the core abbreviated scale are indicated in bold

**Table 5:** Item response analysis of retained all stigma items in the sample from Jordan (n = 108)

All stigma items	Discrimination	Difficulty		
		≥ 1	≥ 2	= 3
Feelings of worthlessness, of having no value *(i)*	1.31	− 0.84	0.06	0.99
**Feeling detached or withdrawn from others *(i)***	1.58	− 1.39	0.02	0.81
Feeling badly treated by family members *(p)*	0.95	− 0.70	0.88	1.92
Feeling badly treated by community members *(p)*	0.95	− 0.24	1.62	3.01
Feeling shame *(i)*	1.02	− 1.28	0.65	1.80
**Blaming yourself for past events *(i)***	1.46	− 1.15	− 0.06	0.62
**Feeling rejected by everybody *(p)***	2.10	− 0.23	1.26	1.98
**Feeling stigma *(p)***	2.85	− 0.02	0.77	1.37
**Wanting to avoid others or hide *(i)***	2.06	− 0.66	0.41	1.00
**Feeling like your family gazes at you like they are blaming you *(p)***	2.04	− 0.07	0.87	1.51
**Feeling like community members gaze at you like they are blaming you *(p)***	1.75	− 0.15	0.94	1.78
Been abandoned/thrown out of your home *(e)*	0.88	− 0.88		
Rejected by your family because of trauma you experienced *(e)*	1.15	0.79		
Rejected by your intimate partner because of trauma you experienced *(e)*	1.01	− 0.12		
Forced to live away from husband because of violence you experienced *(e)*	0.87	1.72		

Suggested items for the core abbreviated scale are indicated in bold

Looking at items that exhibited strong discrimination across both contexts and operated across a range of difficulties, we selected potential core items (items in bold in Table 5). In using MIMIC models to assess if any of these core items had DIF potential, the pathway between country and the following items had a Bonferroni adjusted *p* value (multiplied by 8, i.e., the total number of core items tested) of < 0.05 (see Additional file 1: Table S1): “feeling detached or withdrawn from others”; “blaming yourself for past events”; “feeling rejected by everybody”; and “feeling stigma.” The findings of the subsequent tests of DIF for these items using nested models are presented in Table 6. Only “feeling rejected by everybody” when treated as invariant did not result in a significantly worse fitting model using Bonferroni adjusted *p* value (multiplied by 4, i.e., the total number of constrained to full model comparisons).

**Table 6:** Comparison of nested MIMIC models to assess differential item functioning of core stigma items

	Pathway from country to item				Likelihood ratio test of constrained and full model*
	“Feeling detached”	“Blaming yourself”	“Feeling rejected”	“Feeling stigma”	
Full model	− 0.482 (0.135), 0.000	− 0.488 (0.140), 0.000	0.254 (0.121), 0.035	0.370 (0.115), 0.001	–
Constrained model 1	INVARIANT^a^	− 0.398 (0.137), 0.004	0.363 (0.119), 0.002	0.480 (0.115), 0.000	12.672 (*df* 1), *p* = 0.002
Constrained model 2	− 0.395 (0.132), 0.003	INVARIANT	0.363 (0.122), 0.003	0.480 (0.114), 0.000	12.122 (*df* 1), *p* = 0.002
Constrained model 3	− 0.526 (0.132), 0.000	− 0.532 (0.140), 0.000	INVARIANT	0.316 (0.113), 0.005	4.431 (*df* 1), *p* = 0.1412
Constrained model 4	− 0.553 (0.133), 0.000	− 0.559 (0.137), 0.000	0.167 (0.119), 0.161	INVARIANT	10.453 (*df* 1), *p* = 0.005

^a^The pathway from country to this item was not estimated, as it was treated as invariant in this model

*p values have been multiplied by the number of tests (n = 4) as a Bonferroni correction

We refined the potential core item recommendations to those that did not exhibit DIF. Items that performed well in one of the two settings and/or that exhibited DIF we classified as belonging to an item bank, i.e., the item could be considered for use based on preliminary research in a given setting that assures its relevance (see Table 7). The Cronbach’s alpha for the refined core item stigma scale was 0.82 in Kenya and 0.81 in Jordan. The mean core stigma score was 1.00 in Kenya (standard deviation (sd) = 0.83), lower than in Jordan where the average was 1.23 (sd = 1.09), though the difference was not statistically significant (*p* = 0.10).

**Table 7:** Summary of items to retain, consider, or exclude across contexts for measuring stigma

Items to include	Bank of items to be considered for including depending on relevance to context	Items to exclude
Feeling rejected by everybody^a^ Wanting to avoid other people or hide^a^ Feeling like your family gazes at you like they are blaming you^a^ Feeling like community members gaze at you like they are blaming you^a^ Feeling badly treated by community members Feelings of worthlessness, of having no value Feeling shame Feeling detached or withdrawn Blaming yourself for past events Feeling stigma	Feeling badly treated by family members Been abandoned/thrown out of your home Rejected by your family because of trauma you experienced Rejected by your intimate partner because of trauma you experienced Forced to live away from your husband because of the violence you experienced	Wanting to change the way you dress

^a^Indicates a core item to use in comparisons across contexts

In construct validity analyses, the shortened core stigma scale and felt stigma scale that resulted from the EFA model in Kenya were similarly correlated with functional impairment, depression, impairment specifically due to depression, and disability (Table 8). The core stigma scale had a correlation of rho = 0.51 with depression, but exhibited very little association with disability and functional impairment in Kenya. However, the core and felt stigma scale in Kenya did correlate meaningfully with impairment due to depression specifically as rated on the PHQ-9 (rho = 0.40 and 0.48, respectively). In Jordan, the four-item core stigma scale exhibited a correlation of 0.68 with depression and a lower correlation with functional impairment and disability (0.30 and 0.35, respectively), which were stronger than those observed in the Kenyan sample. Correlations between the core stigma scale and depression in Jordan were equivalent in magnitude to the correlation of these two constructs in Kenya (rho = 0.40). The magnitude of the correlations of the core stigma scale with these variables from the nomological network (functional impairment, disability, and depression) were very similar to the correlations of the refined all-item stigma scale in Jordan.

**Table 8:** Correlation among stigma scales and other variables in the nomological networks in Kenya and Jordan

	Kenya (n = 100)			Jordan (n = 108)	
	Felt stigma	Enacted stigma	Core stigma	All stigma	Core stigma
Depression	0.54	0.21	0.51	0.72	0.68
Impairment from depression^a^	0.48	0.32	0.40	0.39	0.40
Functional impairment	0.09	− 0.11	0.01	0.38	0.30
Disability	0.13	− 0.15	0.07	0.32	0.35

^a^Assessed using the 10th item on the PHQ-9 which asks individuals to indicate how difficult it has been completing work, taking care of things at home or getting along with others due to the problems they endorsed on the PHQ-9: not difficult at all, somewhat, very, or extremely difficult

## Discussion

To enhance monitoring and evaluation of services for survivors of GBV in humanitarian emergencies, we assessed the psychometric properties and construct validity of a measure of sexual violence stigma as adapted for use in two new populations of survivors: Somali refugees in Kenya and Syrian refuges in Jordan. Notably, the sexual violence stigma measure exhibited distinct structures among Somali as compared to Syrian survivors. Similar to findings from eastern DRC where the scale was originally developed, two distinct constructs were identified among Somali women: enacted stigma, i.e., discrimination; and felt stigma, i.e., a combination of internalized and perceived stigma. Among Syrian women seeking GBV services however, one overarching stigma construct was identified. Further, when data from Syrian women was examined as representing a two-factor measure, felt stigma items did not load onto a separate factor from enacted stigma items. Rather, one factor was characterized by internalized stigma items only, and the other was a mixture of perceived and enacted stigma from family and the community. This distinction between felt versus enacted stigma observed in the data from Somali women versus Syrian women is relevant for service providers, as case managers might only have agency to influence felt stigma when working with survivors because enacted stigma cannot necessarily be improved by case management alone. Tracking both manifestations of stigma distinctly can help demonstrate when structural changes and/or programs targeting communities, families, and potential perpetrators of violence (e.g., community mobilization or campaigns to change public attitudes) are necessary to meaningfully improve survivors’ well-being, rather than place the burden of stigma reduction solely on survivors themselves.

There are several possible reasons for the difference in factor structure of the sexual violence stigma measure observed in these two populations. Syrian women reported more recent displacement, more severe symptoms of distress and functional impairment, and a higher frequency of disability than Somali women. These intersecting marginalized identities, such as having a mental disorder or disability, may shape how sexual violence and associated stigma is experienced [57], and in our study, may be one reason we saw poorer item discrimination in the sample of Syrian women as compared to the Somali women. In addition, the sample of Syrian female GBV survivors was also older, more educated, and more likely to be married on average than the Somali sample. It is possible that life stage and the opportunities for different manifestations of stigma to be experienced as more or less important across life stages could explain this difference in structure. For instance, when sexual violence occurs against young Somali women, it was described as being particularly impactful due to how the violation of values and norms around virginity could threaten marriage prospects [58]. This points to a particular need for research on adolescent and school-aged girls versus older women.

Qualitative research with Syrian women has also detailed fears of bringing shame to one’s family in addition to the survivor herself, which can result in social exclusion from communities and families and even possibly honor killing or violent conflict [59]. The possibility of affiliation stigma being experienced by families- for instance, children of survivors experiencing bullying in the community- and cross-cultural differences in how and when affiliation stigma is applied are other potential factors that could affect the latent structures of the measure across contexts. Qualitative research among Somali refugees has also suggested the potential for shame to one’s family resulting from sexual violence [58, 60]. However, at least some Somali participants in these studies expressed that honor killing would not be considered; families were instead described as intervening in support of the survivor [58], balancing providing support to the woman with trying not to lose status within the community [60]. While the role of husbands and families in perpetuating stigma has been described [19, 61] and research has documented the potential for transgenerational affiliative stigma for children born from sexual violence [58, 61], a broader and more nuanced understanding of the role of family as both stigmatizer and stigmatized- and how both the family and woman navigate these competing forces- can serve to both advance stigma measurement and inform interventions across contexts.

Of the individual stigma items assessed, only “wanting to change the way you dress” was dropped from the scale in both settings due to a weak relationship with the underlying stigma factors. This was conceptualized originally as an expression of internalized stigma, reflecting a woman’s desire to hide or not draw attention. It is possible this item did not perform well in part because in both settings women already routinely dressed modestly, e.g. in Jordan wearing a loose fitted *abaya* that fully covered a woman’s arms and legs. The remaining 15 items can be used to measure multiple forms of stigma depending on what is found to be most meaningful and appropriate within a given context. However, several of these items exhibited differential measurement across the two locations and populations. While these items may still have great relevance for tracking change and identifying need within a given population of survivors, this limits their utility in making comparisons across populations or service settings. Service providers therefore need to consider the primary purpose of the monitoring systems and the needs they have for their data.

If service providers work across countries with unique populations of survivors, priority may need to be given to inclusion of core items to facilitate their ability to pool data and make overall conclusions about their programs across settings or to identify programs that may be struggling and in need of greater support. These core items showed consistent evidence of construct validity in their relationship with depression and impairment from depression in both settings. However, functional impairment and disability were only meaningfully related to the core stigma scale in the data from Jordan, not from Kenya where observed associations were negligible. The felt and enacted stigma scale were examined in relation to functional impairment in a prior study of Congolese survivors of sexual violence living within DRC, and a significant association was found [18]. Functional impairment among Somali survivors in Kenya was low overall and may be an indication of the resilience required to successfully flee and/or live within the refugee camp (Dadaab was established in 1991 and thus many residents were born in the camp) and explain the overall lack of association found with stigma in this specific sample.

In addition to practically demonstrating how the same items can be combined in different ways to measure constructs that are appropriate within a given population, the psychometric results also point to areas where additional items might need to be added to create a meaningful measure in a particular context. For instance, if acts of discrimination appear to be a distinct form of stigma experienced in a given place as was the case in Somali sample, it may be that service providers want to add more of these types of items to their measure. These context specific additions could create a scale of greater relevance to the population they serve that can be used to assess more nuanced change in the most meaningful forms of stigma women experience or to make internal program comparisons in change in stigma, such as by case manager. The item bank that has resulted from these analyses can be a starting point for this process in conjunction with qualitative data collection with program beneficiaries and stakeholders.

The results of this study need to be interpreted with consideration of several limitations. The relatively small sample size within each setting limited our ability to engage in more complex analyses, for instance examining how intersectional identities and characteristics might shape the latent structure and psychometrics of the scale. In addition, we were only able to assess construct validity of the developed scales by examining relationships with a few key constructs and were not able to assess criterion validity due to the lack of a gold standard measure being included in our design or dataset, and all measures were assessed via self-report which is subject to potential underreporting due to social desirability bias. The use of audio computer-assisted self-interviewing (ACASI) administered questionnaires could help mitigate this limitation in the future. As we did not assess who the perpetrator was for any experiences of sexual violence, we cannot discern if there are differences in the construct or measurement of stigma arising from non-partner versus partner violence, not the degree to which this could drive differences in measurement observed in the two contexts. How differences in perpetration, including the relationship of the person to the survivor and the conditions in which the event occurred (i.e., during active conflict) shape stigma is an important avenue for future research.

There may be also important forms of sexual violence stigma we are missing, such as anticipated stigma or stigma experienced within a given setting (e.g., healthcare). Anticipated stigma, as well as acts of stigma arising from perpetrators, have emerged as important factors in prior work exploring the psychometrics of a scale of stigma associated with intimate partner violence [35]. Relatedly, with few adolescent girls in our dataset, we were not able to examine stigma experienced in school settings. An important future research direction is to examine how the experience and measurement of sexual violence stigma may vary for adolescent girls versus from older women, particularly by designing a study to include non-service seeking girls as the stigma of experiencing intimate partner violence has been noted to be a critical impediment to seeking support among the adolescent population in particular [62]. An additional important next step is to use frameworks such as “What Matters Most” [63] to continue to uncover how threats to cultural values lead to stigma within additional contexts and populations and to possibly identify commonalities to guide adaptations to improve local meaningfulness and relevance of measures among groups that share common value systems.

## Conclusion

Continued investment in qualitative research on the dynamics of stigma experienced by sexual violence survivors and its use to inform psychometric testing of complex social aspects surrounding GBV in humanitarian settings is needed in order to provide the best prevention and response approaches for women and girls. Providers of GBV response services in humanitarian settings must address different manifestations of stigma to deliver contextually appropriate services for survivors of GBV of the highest quality that are accessible and fulfill a duty of doing no harm to the population they aim to serve. Rigorous and repeated monitoring of program participants to collect indicators that go above and beyond traditional GBV response output metrics, such as numbers of dignity kits distributed and numbers of survivors served, to characterize stigma can inform actionable pivots in the provision of care. The measures of stigma presented here draw on a core set of items that can be efficiently administered and have demonstrated evidence of construct validity from multiple settings, while providing flexibility for the addition of items and recharacterization of different stigma manifestations that may vary across settings. As such, these measures can be implemented directly by case managers as a part of routine M&E services to improve the provision of tailored services to sexual violence survivors, and data collected throughout a program can be aggregated to improve quality of service delivery overall [64].

## Supplementary Information

**Additional file 1:** Assessment of DIF potential among possible core stigma items.

## Data Availability

The datasets generated and/or analyzed during the current study are not currently publicly available. Until such time when ongoing analyses are completed and the dataset becomes publicly available, the data are available from the corresponding author on reasonable request.

## Abbreviations

ACASI: Audio Computer-Assisted Self-Interviewing
CPT: Cognitive Processing Therapy
DRC: Democratic Republic of Congo
DIF: Differential item functioning
EFA: Exploratory factor analysis
GBV: Gender-based violence
IRC: International Rescue Committee
IPV: Intimate partner violence
IRT: Item response theory
KEMRI: Kenya Medical Research Institute
LMIC: Low- and middle-income counties
M&E: Monitoring and evaluation
MIMIC: Multiple Indicators, Multiple Causes Models
PHQ-9: Patient Health Questionnaire
PTSD: Post-traumatic stress disorder
PCA: Principal Components Analysis
SD: Standard deviation
UNFPA: United Nations Population Fund
VSLA: Village Savings and Loan Association
WHODAS 2.0: WHO Disability Assessment Schedule
