# Supplementary material for: Measuring sexual violence stigma in humanitarian contexts: assessment of scale psychometric properties and validity with female sexual violence survivors from Somalia and Syria
Source: Confl Health. 2021 Dec 24;15:96. doi: 10.1186/s13031-021-00431-z (PMC8709979; doi:10.1186/s13031-021-00431-z)
Supplement: Supplementary file 1 — Additional file 1: Assessment of DIF potential among possible core stigma items. [file 13031_2021_431_MOESM1_ESM.docx]

| **Supplemental Table 1. Assessment of DIF potential among possible core stigma items** | | | |
| --- | --- | --- | --- |
| **Stigma Item** | **Stigma Factor on Country** | **Individual Item on Country** | **DIF Potential*** |
|  | β (SE), p-value | β (SE), p-value |  |
| Feeling detached or withdrawn from others | 0.179 (0.153), 0.242 | -0.505 (0.129), <0.001 | Yes |
| Feeling badly treated by community members | 0.064 (0.152), 0.673 | 0.254 (0.143), 0.076 | No |
| Blaming yourself for past events. | 0.178 (0.153), 0.245 | -0.510 (0.135), <0.001 | Yes |
| Feeling rejected by everybody | 0.049 (0.150), 0.744 | 0.340 (0.119), 0.004 | Yes |
| Feeling stigma | 0.016 (0.151), 0.951 | 0.486 (0.112), <0.001 | Yes |
| Wanting to avoid other people or hide | 0.156 (0.153), 0.309 | -0.315 (0.120), 0.009 | No |
| Feeling like your family gazes at you like they are blaming you | 0.102 (0.151), 0.499 | -0.014 (0.139), 0.921 | No |
| Feeling like community members gaze at you like they are blaming you | 0.054 (0.152), 0.725 | 0.279 (0.123), 0.023 | No |
| *p-value less than Bonferroni corrected limit of 0.00625 |  |  |  |
